# Incubation Behavior of the Western Reef Heron (*Egretta gularis*) in Eastern Saudi Arabia: Adaptations to Extreme Thermal Conditions

**DOI:** 10.3390/life15091380

**Published:** 2025-09-01

**Authors:** Monif AlRashidi, Abdulaziz S. Alatawi, Mohammed Shobrak, Mohanad Abdelgadir

**Affiliations:** 1 Department of Biology, Faculty of Science, University of Ha’il, Ha’il 55476, Saudi Arabia; 2 Department of Biology, Faculty of Science, University of Tabuk, Tabuk City 71491, Saudi Arabia; abalatawi@ut.edu.sa; 3 National Centre for Wildlife, Prince Saud Al-Faisal Centre for Wildlife Research, Taif 26618, Saudi Arabia; shobrak@saudibirds.org; 4 School of Natural Sciences, Technology and Environmental Studies, Södertörn University, 14189 Huddinge, Sweden; mohanad.abdelgadir@sh.se

**Keywords:** thermoregulation, incubation posture, nest attendance, harsh environment

## Abstract

The Western Reef Heron (*Egretta gularis*) has a wide geographic distribution, ranging from the coasts of West Africa to Southwest Asia, including the Arabian Peninsula. Despite this extensive range, detailed information on its incubation behavior remains scarce. To address this gap, we investigated the 24 h incubation behavior of Western Reef Herons on Al-Fanateer Island, Eastern Saudi Arabia, during early summer—a period characterized by pronounced diurnal fluctuations in ambient temperature. Using trail cameras and temperature loggers, we found that adults maintained nearly continuous attendance at the nest throughout the day, with incubation coverage exceeding 97% across all two-hour intervals. A slight reduction in nest attendance was observed during nighttime (lowest at 86.8% between 20:00–21:59). Incubating adults exhibited behavioral plasticity in response to ambient temperature: a sitting posture was predominant during cooler periods, while a shading posture was more frequent during peak heat. Incubating adults also adjusted their orientation with the solar angle, actively avoiding southern and western exposures during the hottest parts of the day. Despite substantial variation in ambient temperature, the temperature beneath the clutch ranged from 29.4 to 37.8 °C, which may indicate effective thermoregulation. These findings suggest that a combination of near-continuous nest attendance, posture adjustment, and solar orientation avoidance allows Western Reef Herons to mitigate thermal stress and maintain optimal conditions for embryo and chick development. We recommend long-term monitoring of incubation behavior in this species to further evaluate its adaptability to environmental changes, particularly those driven by climate variability.

## 1. Introduction

Climate change is increasingly intensifying the frequency, severity, and duration of extreme heat events, posing substantial threats to animals’ survival and reproductive viability [1]. Elevated ambient temperatures—particularly within nesting microhabitats—can disrupt the finely tuned thermal conditions essential for embryonic development. These thermal disturbances are associated with a range of detrimental outcomes, including skewed sex ratios and reduced hatching success [2,3]. Such impacts can further lead to physiological dysfunction and reproductive failure, ultimately driving population declines through both direct mortality and sublethal reductions in individual fitness [2,3]. Species with limited adaptive capacity are particularly vulnerable, experiencing steep declines or localized extinctions [4]. In contrast, some taxa persist via microevolutionary adaptations such as genetic selection for heat tolerance or through phenotypic plasticity, enabling rapid behavioral, physiological, or morphological responses to thermal extremes [5,6].

Birds demonstrate considerable adaptability across thermal extremes, from polar to hyper-arid environments [7]. However, species that nest in hot climates without natural shade face significant reproductive challenges, as unattended eggs can reach critical embryonic temperature thresholds within just a few minutes [7,8]. Thus, continuous parental attendance especially during the hottest part of the day is critical to shade eggs and facilitate convective cooling [9,10,11]. However, such sustained attendance imposes significant physiological costs on incubating adults, including dehydration, hyperthermia, and energetic depletion [7,8]. To mitigate lethal thermal exposure, diverse avian lineages have evolved behavioral and physiological thermoregulatory strategies [10,11,12,13,14]; these include prolonged nest attendance to maximize shading [15], evaporative cooling via ventral feather wetting [16], selective nest placement within thermally buffered microhabitats [17,18], and temporary egg-covering with a thin layer of soil or shell fragments during short parental absences [13,19]. While effective, these adaptations entail significant trade-offs, such as reduced foraging time of incubating adults, elevated water requirements, and increased metabolic costs, potentially leading to body mass loss, a condition that can precipitate nest abandonment [7,8].

The Western Reef Heron (*Egretta gularis*) is a medium-sized heron that was previously considered a subspecies of the Little Egret (*Egretta garzetta*) [20]. Hybridization between the two species has been documented, particularly in regions where their ranges overlap [20]. The Western Reef Heron is primarily distributed along coastal areas of Africa and parts of Southwest Asia, and it is increasingly reported as a vagrant in Southern Europe [20,21]. This species displays notable plumage polymorphism, with individuals occurring in white, dark gray, and intermediate morphs that interbreed without restriction [20,21]. Two subspecies are recognized: *E. g. gularis*—found along the West African coast from Mauritania to Gabon. *E. g. schistacea*—which occurs along the coasts of East Africa, the Arabian Peninsula, and parts of Western, Southern, and Southeastern India [20]. This subspecies also migrates to Sri Lanka for the winter [20]. Although the Western Reef Heron is mainly found along the coast, there have been occasional inland sightings, including a notable one in Zambia [20]. It occasionally reaches the Mediterranean and is a rare vagrant in the Western Palearctic, with records from Spain, France, Italy, and several Atlantic islands [20]. Unconfirmed reports exist from the Maldives and Bangladesh. Exceptionally, it has been recorded in the Americas, including sightings in Canada, the Northeastern United States, the Caribbean, and coastal Brazil [20].

*E. gularis* is a breeding resident along the coasts and islands of Arabia, with confirmed breeding across all states [22,23,24,25], The total Arabian population is estimated at around 3000 pairs, primarily inhabiting saline coastal environments such as tidal mudflats, mangroves, and sandbars [23]. It also makes use of inland wetlands and artificial habitats [23]. The Western Reef Heron nests in colonies, where the breeding season lasts from February to August [22,23]. Nests are pads of twigs, commonly of dry halophytes, and sometimes a little seaweed or algae, often on predator-free islands that offer suitable vegetation, including *Avicennia marina*, *Arthrocnemum* spp., *Seidlitzia* spp., and *Euphorbia fractiflexa* [23]. Nests are constructed on the ground, in bushes, on cliffs, or on artificial structures such as buoys and shipwrecks. Colony sizes vary widely, ranging from a few pairs to more than 150 [22,23]. Clutches typically consist of 3–4 laid eggs, and the incubation begins from the moment the first egg is laid by both parents over a period of 26–28 days. The chicks fledge approximately seven weeks after hatching [22,23].

Despite the wide range of *E. gularis*, detailed knowledge regarding its incubation behavior remains limited. To help bridge this knowledge gap, our study was conducted in the Eastern Province of Saudi Arabia during early summer—a period marked by sharp fluctuations in ambient temperature throughout the day. This study aimed to quantify nest attendance patterns and behavioral thermoregulation during incubation, with a focus on the mechanisms employed by incubating adults to protect eggs and chicks from extreme temperatures.

## 2. Materials and Methods

### 2.1. Study Site

**Figure 1:**
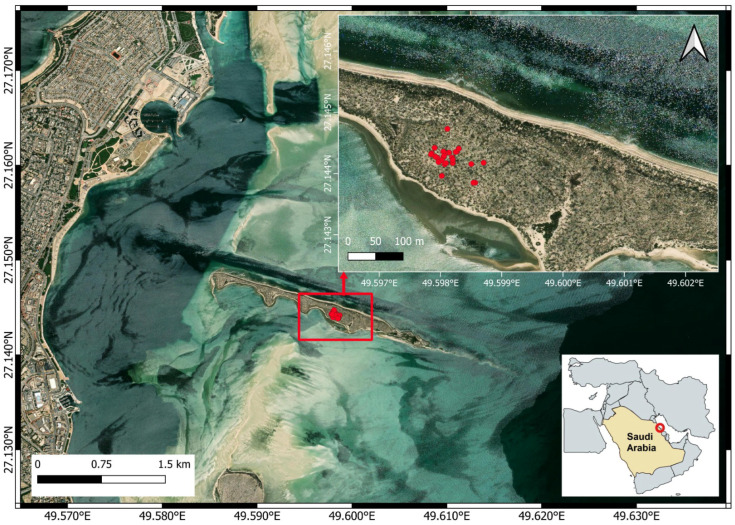
Map of Al-Fanateer Island, Eastern Saudi Arabia, showing the locations of Western Reef Heron nests marked by red dots. The red circle shows the geographical location of the study area. Map created using QGIS v. 3.40, an open-source software (QGIS Development Team, 2022).

Al-Fanateer Island is a manmade island, covering approximately 190,000 m^2^, created in the 1980s using sand and rock debris dredged from the seafloor during the construction of a canal to facilitate ships’ passage to the port of Jubail Industrial City. The island lies approximately 1.5 km northeast of Jubail Industrial City, in the Eastern Province of Saudi Arabia. Designated as a bird sanctuary, the island is managed by the Royal Commission for Jubail and Yanbu. It is characterized by dense vegetation, primarily composed of halophytic shrubs, with *Salsola vermiculata* and *Suaeda baryosma* being the dominant species (Figure 1). The study site is characterized by a desert climate, with extremely hot summers, mild to cool winters, and minimal rainfall. In summer, daytime temperatures generally reach around 45 °C [22]. The area experiences arid conditions, limited cloud cover, and notable temperature variations between day and night, as well as across seasons.

### 2.2. Data Collection

This study was conducted during the breeding season from 30 May to 4 June 2016. For each nest, nest height, nest diameter, and clutch size were recorded. Each nest was photographed, and its geographic coordinates were documented using a handheld Garmin eTrex GPS unit (Garmin^®^; Olathe, KS, USA). To monitor nest attendance behavior and detect any disturbances or threats to incubating adults and their clutch, a Moultrie M-880i Gen 2 trail camera (Moultrie^®^; Birmingham, AL, USA) was installed approximately 1.5 m from each of seven nests (Table 1). The nests were randomly selected by entering the identification numbers of all nests into Microsoft Excel and using the RANDBETWEEN function to select seven. The camera captured one image per minute continuously over a 24 h interval, resulting in a total of 10,080 images. Equipped with infrared sensors, the camera recorded both diurnal and nocturnal activity. Installation took less than 10 min, and adult birds typically returned to their nests within a few minutes. The camera also featured a built-in data logger that recorded ambient temperature and displayed it on each image. In addition, a DS1922L-F5 Thermochron iButton data logger (iButtonLink Technology^®^; Whitewater, WI, USA) was placed beneath the clutch to record temperature at one-minute intervals.

### 2.3. Data Analysis

A 24 h recording served as the unit of analysis for each nest (n = 7), with each recording segmented into twelve 2 h intervals. Following [9,11,26], two primary behavioral variables were quantified for each interval: (1) nest attendance, defined as the proportion of time the nest was incubated by either parent, and (2) orientation, defined as the proportion of time the incubating adult faced each of the four cardinal directions (north, south, east, and west).

Additionally, three distinct incubation postures were identified and quantified: (1) sitting—the percentage of time the parent maintained full body contact with the eggs/chicks while sitting tightly on the nest; (2) shading—the percentage of time the parent shaded the eggs/chicks by drooping its wings over the nest without making full body contact; and (3) standing—the percentage of time the parent stood on the nest without any physical contact with the eggs/chicks. Ambient temperature and temperature beneath the clutch were averaged for each two-hour interval.

To investigate the influence of ambient temperature on nest attendance, linear mixed-effects models (LMMs) were used [9,27]. Nest identity was included as a random effect, since incubation behavior is not independent across time within each nest. The model incorporated a random intercept for each nest, with time interval included as a fixed factor and ambient temperature modeled as a second-degree orthogonal polynomial covariate to capture potential non-linear relationships [28]. An interaction term between time interval and temperature was also included, as the effects of temperature may vary across the day [9].

Separate LMMs were constructed to assess the relationship between ambient temperature and the three incubation postures (sitting, shading, and standing). Fixed effects in the initial models included the following: time interval (factor), ambient temperature (second-degree orthogonal polynomial covariate), incubation posture (factor with three levels), and their three-way interaction. Nest identity was again included as a random intercept. To evaluate the effect of sunlight direction on adult orientation during incubation, additional LMMs were fitted with nest identity as a random effect [15]. Fixed effects in these models included cardinal direction (factor with four levels: north, south, east, west), time interval (factor), ambient temperature (second-degree orthogonal polynomial covariate), and their three-way interaction. All response variables were arcsine square-root transformed to meet assumptions of normality. Additional covariates potentially affecting incubation behavior—nest height, nest diameter, clutch size, clutch type (a two-level factor: eggs or chicks), and the distance to the nearest nest—were included in the initial models but were excluded from the final models after being found non-significant during model selection (*p* ≥ 0.060). Model fitting was performed using maximum likelihood estimation, and model selection was based on likelihood ratio tests using the ANOVA function [29]. All statistical analyses and figure generation were performed using R version 4.5.1 [30].

## 3. Results

### 3.1. Nest Characteristics

A total of 26 nests were documented, all located in the central part of the island (Figure 1). Each nest was constructed atop Mediterranean saltwort (*Salsola vermiculata*). Clutch sizes ranged from one to four eggs or chicks, with a median of three. Nest height ranged from 30 to 73 cm (median = 60 cm), and nest diameter varied from 30 to 55 cm (median = 40 cm). The distance to the nearest nest ranged from 1.5 to 57.5 m (median = 12.82 m).

### 3.2. Temperature Variations

**Figure 2:**
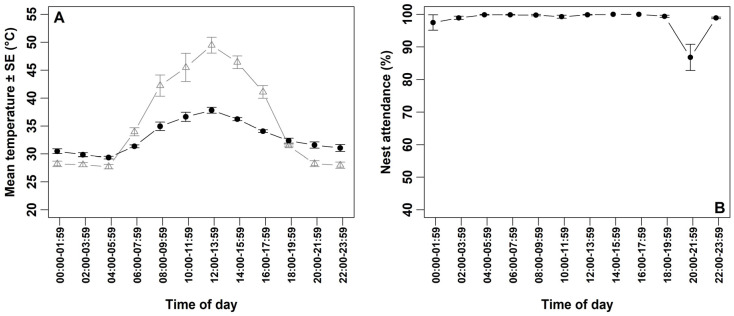
(**A**) Mean ambient temperature (±SE) recorded by camera-integrated data loggers positioned ~1.5 m from each nest (open triangles, n = 7 nests) and mean of temperature (±SE) recorded beneath the clutch by Thermochron iButton data loggers (filled circles, n = 7 nests). (**B**) Percentage of time spent by Western Reef Herons attending their nests in relation to ambient temperatures across the two-hour intervals (n = 7).

Ambient temperature exhibited substantial diurnal variation (Figure 2A; Table 2). The minimum recorded temperature was 25 °C during the early morning hours (04:00–05:59) on 2 June, while the maximum reached 58 °C during the hottest two-hour interval (12:00–13:59) on the same day. The average ambient temperature during the coldest two-hour interval (04:00–05:59) was 27.73 ± 0.40 °C, while during the hottest interval (12:00–13:59), it reached 49.47 ± 1.42 °C (Figure 2A; Table 2). The temperature beneath the clutch ranged from 29.36 to 37.83 °C (Figure 2A; Table 2).

### 3.3. Incubation Behaviors

#### 3.3.1. Nest Attendance

Overall nest attendance was consistently high, with an average of 98.43 ± 0.57% (n = 7 nests), with each nest attending for more than 95% of the 24 h period (Table 1). Attendance remained above 97% across all two-hour intervals, except between 20:00 and 21:59, when mean nest attendance decreased to 86.79 ± 4.03% (n = 7 nests) (Figure 2B; Table 2). During this interval, the average ambient temperature was 28.28 ± 0.53 °C, while the temperature beneath the clutch had an average of 31.60 ± 0.56 °C (n = 7 days) **(**Figure 2B; Table 2). Although a brief interruption in incubation behavior was observed during this period, still images from the camera did not indicate any apparent external threats. Ambient temperature alone did not significantly affect overall nest attendance; however, its interaction with time of day had a significant effect (Table 3).

#### 3.3.2. Incubation Postures

**Figure 3:**
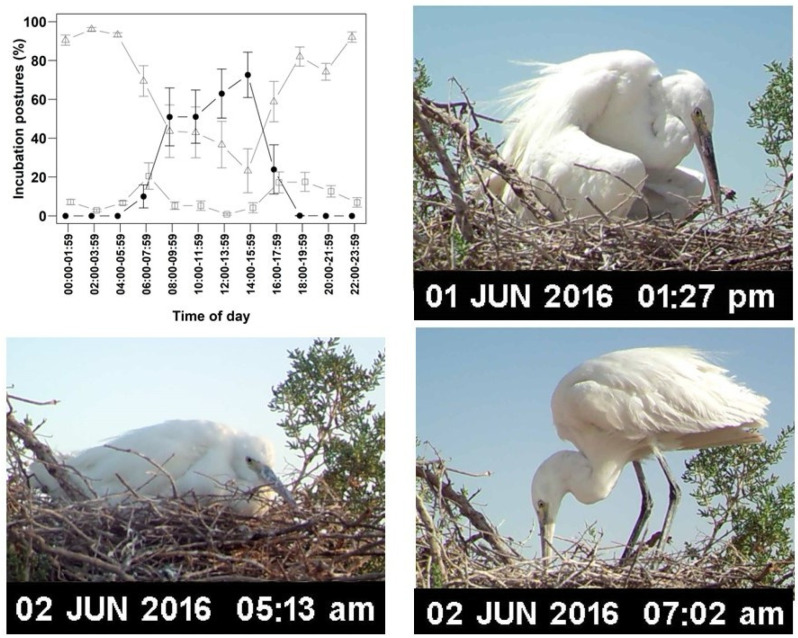
**Top left**: Incubation postures (%) of Western Reef Herons during two-hour intervals (n = 7 nests); shading posture: filled circles; sitting posture: open triangles; standing posture: open squares. **Top right**: shading posture, where the parent shades the eggs/chicks by drooping its wings over the nest without making full body contact. **Bottom left**: sitting posture, where the parent maintains full body contact with the eggs/chicks while sitting tightly on the nest. **Bottom right**: standing posture, where the parent stands on the nest without any physical contact with the eggs/chicks.

Ambient temperature significantly influenced incubation posture (Figure 3; Table 3). Sitting posture’s prevalence increased with decreasing ambient temperature, whereas shading posture increased with rising ambient temperature (Figure 3; Table 3). Standing posture was most prevalent for three two-hour intervals—06:00–07:59, 16:00–17:59, and 18:00–19:59—exceeding 17% in each. The corresponding average ambient temperatures during these intervals were 33.98 ± 0.72 °C, 41.10 ± 1.13 °C, and 31.45 ± 0.18 °C, respectively, while the average temperatures beneath the clutch were 31.36 ± 0.25 °C, 34.10 ± 0.24 °C, and 32.38 ± 0.44 °C (n = 7 nests) (Figure 3; Table 2).

#### 3.3.3. Orientation of Incubating Parents

**Figure 4:**
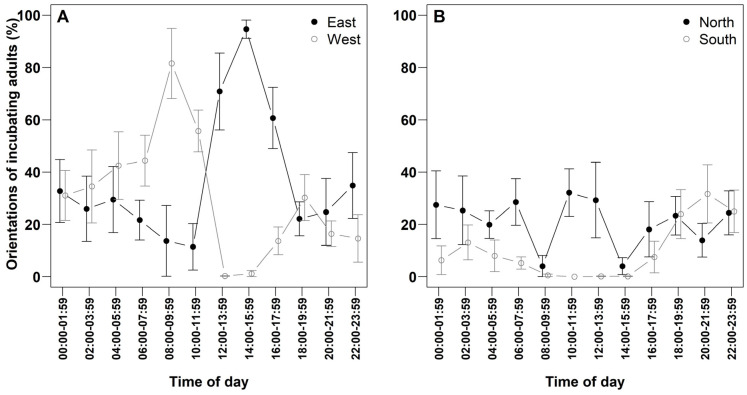
Orientation of incubating adult Western Reef Herons (%), recorded over two-hour intervals (n = 7 nests). (**A**) Proportion of individuals oriented toward east and west. (**B**) Proportion oriented towards north and south. Values are presented as means ± standard error (SE).

Adult orientation during incubation displayed temporal patterns potentially influenced by solar position (Figure 4; Table 4). In the morning, individuals predominantly faced west. Between 10:00 and 11:59, west was the predominant orientation, followed by north. During the hottest interval (12:00–15:59), orientation shifted towards east more frequently than north. Notably, fewer than 1% of individuals faced south or west during the hottest interval (12:00–13:59). During the night (18:00–03:59), incubating adults showed no clear directional preference, with all orientations occurring at frequencies below 35%.

### 3.4. Parental Care

Camera recordings confirmed biparental presence at nests, particularly those containing chicks, where one parent was observed delivering food while the other remained at the nest (Table 1). In contrast, no changeovers—instances where one parent relieved the other—were recorded at nests containing only eggs.

## 4. Discussion

This study identifies three primary behavioral strategies employed by Western Reef Herons to mitigate thermal stress during incubation under conditions of pronounced ambient temperature fluctuations: (1) high nest attendance, (2) dynamic postural adjustments, and (3) solar-avoidant orientation. Collectively, these strategies contribute to effective thermoregulation of the clutch [11,15].

The nest attendance rate of Western Reef Herons was found to be markedly higher than that reported for ground-nesting species such as the Kentish Plover (*Charadrius alexandrinus*), Saunders’s Tern (*Sternula saundersi*), Lesser Crested Terns (*Thalasseus bengalensis*), Black-winged Stilt (*Himantopus himantopus*), and Cream-colored Courser (*Cursorius cursor*) that breed in the region [9,10,11,15,31]. This disparity may be partially attributed to differences in nesting substrate. Western Reef Herons predominantly utilize Mediterranean saltwort as a nesting platform, which provides substantial thermal insulation from ground heat. This insulation mitigates the thermal stress experienced by the incubating adult, facilitating prolonged nest attendance periods. In contrast, ground-nesting species are subjected to increased radiant heat flux from the soil surface [8,32,33], which may necessitate temporary nest absences, especially during periods when ambient temperatures fall within the optimal range for egg development, to allow adults to replenish energy reserves and maintain physiological homeostasis. Additionally, during the morning and late afternoon, some ground-nesting species cover their eggs with a thin layer of soil or shell fragments when temporarily leaving the nest [19,34]. This behavior enhances egg camouflage and helps maintain egg temperatures within optimal thermal thresholds for embryonic development [19,34]. In contrast, the Western Reef Heron does not engage in such egg-covering behavior, and any absence from the nest may lead to a drop in egg temperature.

A comparison with the Eurasian Spoonbill (*Platalea leucorodia*), which breeds synchronously with the Western Reef Heron on the same island [35], indicated higher nest attendance in the latter species. However, this result should be interpreted with caution, as the incubation data for *P. leucorodia* were derived from a single nest that ultimately failed to produce any fledglings, thereby limiting the reliability and generalizability of the comparison. Moreover, still-image camera monitoring revealed frequent incubation changeovers between mates in the all ground-nesting species studied in the region [11,15,31]. In contrast, no such incubation shifts were observed for the Western Reef Heron, even though the presence of both parents at the nest was recorded by the cameras during the chick-rearing period. The absence of incubation changeovers in this species may be explained by the superior thermal buffering provided by the saltwort nesting environment. Conversely, ground-nesting species frequently alternate incubation duties to cope with thermal stress and engage in thermoregulatory behaviors such as wetting their belly feathers to cool the eggs.

In addition to nest attendance, solar-avoidant orientation was consistently observed in incubating Western Reef Herons. Adults regularly adjusted their body position to shield eggs from direct solar radiation, a behavior similarly reported in other arid-zone species [11,15,31,35]. Typically, individuals oriented westward during the morning, gradually rotating clockwise to an eastward orientation by evening. This behavior likely serves to maximize shading of the clutch throughout the day, thereby mitigating the risk of thermal overload. An alternative explanation suggests that by avoiding direct sunlight, incubating adults reduce glare, which may otherwise impair visual acuity and compromise their ability to detect potential predators [11].

Thermal data collected from beneath the clutches indicate that Western Reef Herons effectively maintained incubation temperatures within a physiologically safe range, spanning 29.4 °C to 37.8 °C. This is consistent with known thermal thresholds for avian embryonic development, where prolonged exposure below 25 °C or above 44 °C is associated with developmental arrest or mortality, respectively [7,14]. Incubating adults elevated clutch temperatures during cooler periods by sitting directly on the eggs and reduced heat gain during peak ambient temperatures through shading behaviors. These findings agree with similar thermoregulatory patterns documented in other desert-breeding birds, such as the Black-winged Stilt, which maintains clutch temperatures between 31 °C and 40 °C [11], and the Cream-colored Courser, which regulates clutch temperatures within a broader range of 26.5 °C to 40.7 °C [31].

This 6-day study period represented approximately 20% of the incubation phase (26–28 days) and was conducted during early summer, a period characterized by pronounced diurnal temperature fluctuations. Given that the breeding season of the Western Reef Heron extends from February to August, we recommend future studies to collect more extensive data across the full incubation period and at different stages of clutch development.

## 5. Conclusions

This study investigated the incubation behavior of the Western Reef Heron in Eastern Saudi Arabia, a region characterized by substantial diurnal fluctuations in ambient temperature. It highlights the behavioral strategies employed by incubating adults to mitigate heat stress and ensure the protection of their clutch. By documenting these adaptive responses, this study contributes to a better understanding of how this species copes with thermal extremes. In light of rising global temperatures driven by climate change, continued behavioral monitoring of this population, as well as comparative studies across different geographic regions, will be essential for assessing the phenotypic plasticity of the Western Reef Heron and its potential resilience to environmental change.

## Figures and Tables

**Table 1:** Dates and time data collection of incubation, clutch size and type, nest attendance (%) over a 24 h period for each nest, and total duration of simultaneous parental presence in monitored nests (n = 7 nests).

Nest ID	Acquired Date		Clutch Size	Clutch Type	Nest Attendance (%) Over a 24-Hour Period	Total Minutes both Parents Were Simultaneously on the Nest
	Start	End				
WRH1	30 May, 12:00	31 May, 11:59	2	One egg/one chick	100.00	7
WRH2	30 May, 12:00	31 May, 11:59	4	Eggs	98.40	-
WRH3	30 May, 12:00	31 May, 11:59	4	One egg/three chicks	98.89	10
WRH4	1 June, 18:00	2 June, 17:59	3	Eggs	98.13	-
WRH5	1 June, 10:00	2 June, 09:59	2	Eggs	98.06	-
WRH6	1 June, 18:00	2 June, 17:59	3	Chicks	100.00	3
WRH7	3 June, 10:00	4 June, 09:59	4	Eggs	95.56	-

**Table 2:** Means and standard errors of ambient temperatures, temperatures beneath the clutch, nest attendance percentages, and proportions of incubation postures recorded over 2 h periods (n = 7 nests).

Time of Day	Ambient Temperature		Temperature Beneath the Clutch		Nest Attendance %		Incubation Postures %					
							Sitting		Standing		Shading	
	Mean	±SE	Mean	±SE	Mean	±SE	Mean	±SE	Mean	±SE	Mean	±SE
00:00–01:59	28.24	0.48	30.49	0.43	97.62	2.36	90.48	2.66	7.14	1.37	0.00	0.00
02:00–03:59	28.09	0.38	29.87	0.36	98.93	0.51	96.07	0.90	2.86	0.63	0.00	0.00
04:00–05:59	27.73	0.40	29.36	0.24	99.88	0.12	93.21	1.07	6.67	1.03	0.00	0.00
06:00–07:59	33.98	0.72	31.36	0.25	99.88	0.12	69.40	7.90	20.48	6.79	10.00	5.90
08:00–09:59	42.24	1.90	34.95	0.74	99.76	0.24	43.57	13.52	5.24	1.96	50.95	14.91
10:00–11:59	45.50	2.53	36.64	0.83	99.29	0.46	42.98	13.10	5.24	2.38	51.07	13.70
12:00–13:59	49.47	1.42	37.83	0.54	100.00	0.00	36.19	12.01	0.83	0.55	62.98	12.62
14:00–15:59	46.42	1.14	36.23	0.28	100.00	0.00	23.21	11.30	4.17	2.58	72.62	11.69
16:00–17:59	41.10	1.13	34.10	0.24	100.00	0.00	58.81	10.37	17.26	5.40	23.93	12.67
18:00–19:59	31.45	0.18	32.38	0.44	99.64	0.35	82.02	4.91	17.50	4.92	0.12	0.12
20:00–21:59	28.28	0.53	31.60	0.56	86.79	4.03	74.17	4.35	12.62	2.91	0.00	0.00
22:00–23:59	28.00	0.57	31.07	0.63	98.93	0.24	92.02	2.65	6.90	2.45	0.00	0.00

**Table 3:** Minimal mixed-effects models were used to analyze nest attendance (%), the type of nest attendance %, and the orientation of incubating adults (%) (n = 7 nests). Ambient temperature was included in the models as a second-degree orthogonal polynomial. Degrees of freedom (*df*) are presented as numerator and denominator, respectively. A dash (–) indicates that the variable was either excluded during model selection or not included in the initial model.

Explanatory Variables	Response Variables								
	Nest Attendance %			Incubation Postures %			Orientation of Incubating Adults %		
	*df*	*F*	*p*	*df*	*F*	*p*	*df*	*F*	*p*
Ambient temperature	–	–	–	2 + 237	112.93	<0.001	2 + 314	5.19	<0.001
Incubation postures	–	–	–	2 + 237	207.97	<0.001	–	–	–
Ambient temperature × Incubation postures	–	–	–	4 + 237	89.61	<0.001	–	–	–
Ambient temperature × Time interval	2 + 75	15.40	<0.001	2 + 84	3.42	<0.001	–	–	–
Cardinal direction	–	–	–	–	–	–	3 + 314	12.20	<0.001
Ambient temperature × Cardinal direction	–	–	–	–	–	–	6 + 314	4.03	<0.001
Cardinal direction × Time interval	–	–	–	–	–	–	4 + 314	4.84	<0.001

**Table 4:** Means and standard errors of the orientation percentages of incubating adults during 2 h periods (n = 7 nests).

Time of Day	Orientation of Incubating Adults %							
	East		West		North		South	
	Mean	±SE	Mean	±SE	Mean	±SE	Mean	±SE
00:00–01:59	32.74	12.01	31.07	9.59	27.50	12.96	6.31	5.52
02:00–03:59	25.95	12.47	34.52	13.96	25.36	13.13	13.10	6.65
04:00–05:59	29.52	12.60	42.50	12.92	19.88	5.27	7.98	6.02
06:00–07:59	21.67	7.62	44.40	9.71	28.57	8.92	5.24	2.33
08:00–09:59	13.69	13.55	81.55	13.37	4.05	4.05	0.48	0.48
10:00–11:59	11.43	8.88	55.71	7.96	32.14	9.07	0.00	0.00
12:00–13:59	70.36	14.71	0.24	0.15	29.29	14.45	0.12	0.12
14:00–15:59	94.64	3.47	1.19	1.19	4.05	3.25	0.12	0.12
16:00–17:59	60.71	11.68	13.69	5.29	18.10	10.56	7.50	6.04
18:00–19:59	22.14	6.48	30.24	8.78	23.33	7.40	23.93	9.39
20:00–21:59	24.76	12.80	16.43	4.86	13.93	6.41	31.67	11.12
22:00–23:59	34.88	12.61	14.64	9.06	24.40	8.40	25.00	8.13

## Data Availability

The original contributions presented in this study are included in the article.
